# Weak Localization in Polycrystalline Tin Dioxide Films

**DOI:** 10.3390/ma13235415

**Published:** 2020-11-28

**Authors:** Vitaly Ksenevich, Vladimir Dorosinets, Dzmitry Adamchuk, Jan Macutkevic, Juras Banys

**Affiliations:** 1 Faculty of Physics, Belarusian State University, Nezalezhnastsi av.4, 220030 Minsk, Belarus; ksenevich@bsu.by (V.K.); dorosinets@bsu.by (V.D.); AdamchukDV@bsu.by (D.A.); 2 Center for Physical Science and Technology, Sauletekio av. 3, 01100 Vilnius, Lithuania; 3 Faculty of Physics, Vilnius University, Sauletekio av. 9, 10222 Vilnius, Lithuania; juras.banys@ff.vu.lt

**Keywords:** tin dioxide films, X-ray diffraction, electrical transport, magnetoresistance, weak localization

## Abstract

The electrical and magnetotransport properties of nanocrystalline tin dioxide films were studied in the temperature range of 4–300 K and in magnetic fields up to 8 T. SnO_2−δ_ films were fabricated by reactive direct current (DC) magnetron sputtering of a tin target with following 2 stage temperature annealing of synthesized samples. The nanocrystalline rutile structure of films was confirmed by X-ray diffraction analysis. The temperature dependences of the resistance *R*(*T*) and the negative magnetoresistance (MR) were explained within the frame of a model, taking into account quantum corrections to the classical Drude conductivity. Extracted from the *R*(*T*) and *R*(*B*) dependences electron dephasing length values indicate the 3D character of the weak localization (WL) in our samples.

## 1. Introduction

Tin dioxide belongs to the family of transparent conducting oxides such as ZnO, In_2_O_3_, ITO (indium tin oxide), etc. and attracts tremendous attention among scientists and manufacturers of optoelectronic devices due to the unique coexistence of high conductivity and optical transparency in the visible range of electromagnetic spectrum [1,2,3]. Combination of the excellent electrical and optical properties of tin dioxide are exploited for such applications as transparent electrodes in solar cells, flat-panel displays, touch-sensitive control panels, coatings for energy-conserving windows in ovens and antifogging windows in airplanes, light-emitting diodes, UV sensors, highly active photocatalysts [3,4,5,6,7,8,9,10]. The high sensitivity of tin dioxide electrical conductivity due to the interaction processes of its surface with the gas molecules is utilized for the fabrication of gas, chemical and humidity sensors [11,12]. Due to the large variety of applications of conductive tin dioxide films, understanding the influence of different types of dopants on their electrical properties is of great importance. The effects of N, P, As, Sb, F and other dopants providing *n*-type of SnO_2_ conductivity were studied both experimentally and theoretically from first-principles calculations [13,14,15,16]. Besides impurities, the high value of conductivity of tin dioxide can be induced by unintentional doping of native point defects (for example, Sn interstitials and O vacancies) during the synthesis process [14,17,18]. Due to the unintentional high concentration of intrinsic *n*-type point defects, it is difficult to provide *p*-type of conductivity of the tin dioxide films. For example, doping of SnO_2_ by Cu, Al and In induces a decrease of intrinsic *n*-type conductivity as a result of the compensation effect [19]. Depending on the crystalline structure and the dopants concentration, different charge transport mechanisms can dominate in SnO_2_ films: hopping conductivity [20,21,22], thermal activation [23], tunneling through the grain boundaries [24,25]. Quantum corrections to the conductivity due to weak localization (WL) and electron–electron interaction effects (EEI) can be observed in highly doped tin dioxide films at low temperatures [15,26,27,28,29]. WL and EEI effects are usually observed in disordered metals under the condition of a high probability of elastic scattering at impurity centers when the probability of backscattering of charge carriers increases [30].

It should be noted that the controlled variation of oxygen vacancies concentration for providing the necessary value of conductivity of the non-stoichiometric tin dioxide films SnO_2−δ_ during synthesis is a quite difficult technological task. We recently developed the method of fabrication of tin oxide films with different phase and stoichiometric compositions, which allows the synthesis of the samples with varied in wide range conductivity magnitude [31]. As a result, different models can be exploited to explain the features of the electrical properties of our samples. In this study, we focused our efforts on the investigation of the electrical and magnetotransport properties of the most conductive SnO_2−δ_ films in which quantum corrections to the classical Drude conductivity due to weak localization and electron–electron interaction effects were observed.

## 2. Materials and Methods

Nonstoichiometric SnO_2−δ_ films were fabricated by reactive DC magnetron sputtering in argon–oxygen plasma of tin (purity is 99.999%) onto glass substrates with subsequent thermal oxidation of the formed layers in air [31]. The total pressure during the deposition process was in the range of 5–10 Pa. The oxygen content in the argon–oxygen plasma was about 1 vol %. The 2 stage heat treatment process with the isothermal annealing at 200 °C (near the melting temperature of Sn) followed by high-temperature annealing at the temperatures within the range of 300–450 °C was used in order to fabricate conductive and transparent tin oxide films [31]. All films were deposited during the same time (30 min) and with the same target-substrate distance (3 cm).

The phase composition of SnO_2−δ_ films was determined by X-ray diffraction (XRD) using an Ultima IV diffractometer (RIGAKU, Tokyo, Japan) in a parallel beam configuration with monochromatic CuKα copper radiation (0.154178 nm) and a D/teX high-speed X-ray detector.

The optical transmission and reflection spectra of films deposited on glass substrates were registered in the spectral range of 200–3000 nm using a PHOTON RT UV-vis-MWIR spectrophotometer (EssentOptics Ltd., Minsk, Belarus). The spectral resolution of the device is 1.2 nm. PHOTON RT UV-vis-MWIR spectrophotometer was also used to estimate the thickness of the samples by the ellipsometric method. All samples have a thickness of about 100 nm.

Scanning electron microscope (SEM) images of the samples were recorded using LEO-1455 VP SEM (Carl Zeiss, Oberkochen, Germany).

The temperature and magnetic field dependences of the resistance were measured using closed-cycle helium refrigerator CFHF Cryogenics Ltd. (London, UK) in the temperature range of 4–300 K and in magnetic field up to 8 T. Electrical measurements were carried out using the standard 4 probe method. Samples were rectangular shaped with the size of about 10 × 5 mm. The current and the voltage were measured by 6430 SourceMeter (Keythley Instruments, Cleveland, OH, USA). The charge carrier’s concentration in the films was determined by means of Hall effect measurements. The Hall voltage was measured using 2182 Nanovoltmeter (Keythley Instruments, Cleveland, OH, USA). Contacts were made by Ag paint.

## 3. Results and Discussion

**Figure 1:**
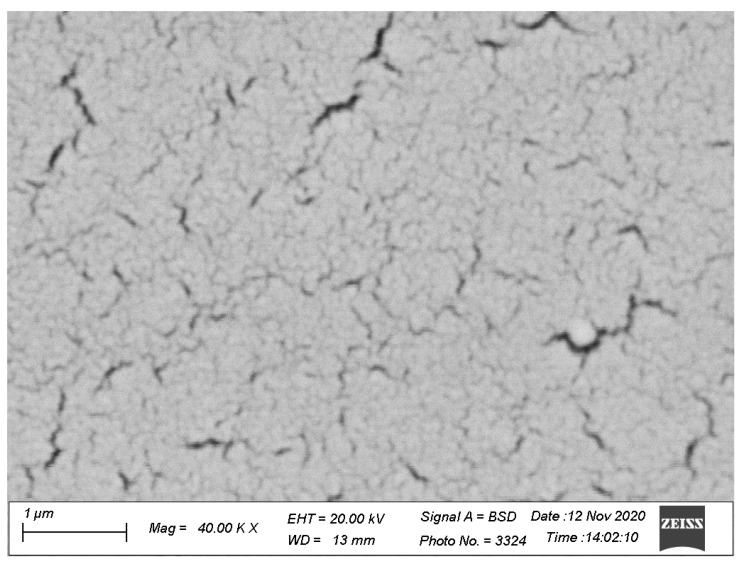
SEM image of the SnO_2−δ_ film deposited on glass substrates by reactive magnetron sputtering of tin in the argon–oxygen atmosphere (with oxygen content of about 1 vol %) with the following annealing in air at 450 °C.

SEM images of the polycrystalline SnO_2−δ_ films are shown in Figure 1. The SEM image shows cracks in the film, which, however, does not break its continuity. The actual resolution of the SEM microscope was about 30–40 nanometers, and crystallites were not resolved in the SEM images. Therefore, the data of XRD analysis were used for the estimation of the grain sizes.

**Figure 2:**
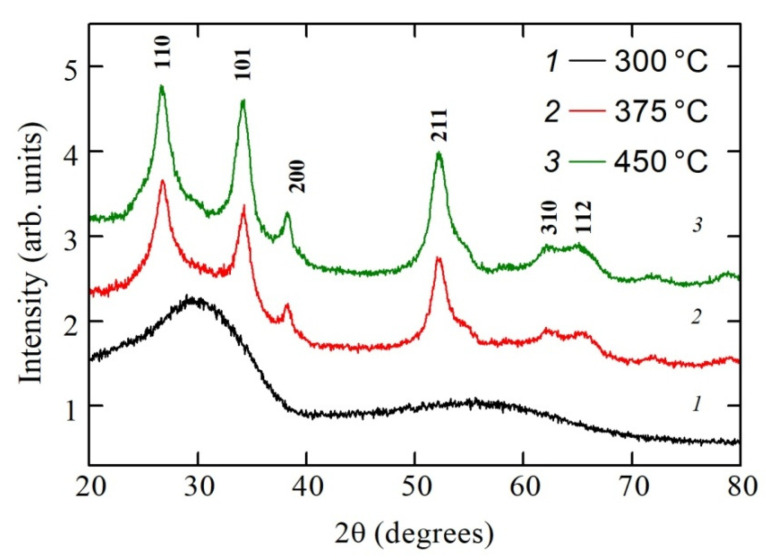
XRD patterns of the SnO_2−δ_ films deposited on glass substrates by reactive magnetron sputtering of tin in the argon–oxygen atmosphere (with oxygen content of about 1 vol %) with the following annealing in air.

The XRD spectra of the polycrystalline SnO_2−δ_ films are shown in Figure 2. Unlike the films fabricated by magnetron sputtering in Ar plasma with the following high-temperature annealing in air and consisted both of SnO and SnO_2_ phases, samples synthesized by DC reactive magnetron sputtering in argon–oxygen plasma (with a low oxygen content of about 1 vol %) consisted of mainly tin dioxide phase [31]. Annealing of the film at 300 °C (we refer to this film as sample A) led to the formation of the amorphous SnO_2_ structure. Only wide bands are visible in the XRD spectra of this sample, as one can see in Figure 2. The diffraction peaks clearly observed around 26.6°, 33.9°, 38°, and 52° for the films annealed on the 2nd stage of the heat treatment procedure at the temperatures 375 °C and 450 °C (we refer to these films as samples B and C, respectively) can be assigned to the (110), (101), (200) and (211) planes of the SnO_2_ tetragonal rutile structure.

Broad peaks indicate the formation of polycrystalline films with grain sizes in the nanoscale range. Estimation of the crystallites sizes oriented along with the (110), (101) and (211) planes was done from the full-width at half-maximum (FWHM) intensity of the observed peaks using the Debye–Scherrer equation in the form [32]:

*D* = *Kλ*/*β*cos*θ*, (1)

where *K* is the shape factor which is usually equal to 0.89, *λ* is the radiation wavelength of CuKα equal to 0.154178 nm, *θ* is the Bragg diffraction angle, and *β* is the full-width at half maximum (FWHM) of diffraction peak. Calculations give values of about 5–7 nm for average grain size. The instrument broadening was not included in the estimation due to its negligible value (0.08 degree) in comparison with the width of the XRD peaks for our polycrystalline samples.

Wide peaks due to X-ray diffraction on (310) and (112) planes of the SnO_2_ tetragonal rutile structure were also detected for samples B and C. It should be noted that increasing oxygen content during the reactive magnetron sputtering process up to a value of about 2 vol % induces the formation of amorphous tin oxide films after following the heat treatment procedure regardless of the annealing temperature [31,33].

**Figure 3:**
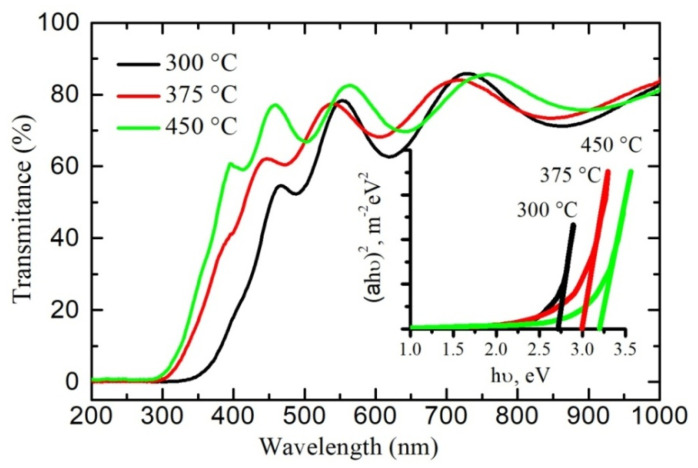
Optical transmission spectra of the SnO_2−δ_ films. Inset shows the dependence of the parameter *(αhν)*^2^ on photon energy *hν*, where *α* is the adsorption coefficient.

The UV-visible transmission spectra of the polycrystalline SnO_2−δ_ films fabricated using reactive magnetron sputtering with 1% oxygen content in argon–oxygen plasma are shown in Figure 3. All samples are characterized by the optical transparency of about 60–80% in the visible range of the electromagnetic spectrum. The optical energy band gaps *E_g_* for the films annealed at different temperatures were determined using conventional Tauc expression for direct-bandgap materials [34]:

*αhν* = *A*(*hν* − *E_g_*)^1/2^, (2)

where *α* is the optical adsorption coefficient, *hν* is the incident photon energy, *A* is a constant, and *E_g_* is a optical energy band gap.

The Swanepoel’s envelope method was employed for estimation of the absorption coefficient *α* and the film thickness *d* from the transmittance spectra [35]. The calculation procedure is described in detail in [31].

The plots (*αhν*)^2^ against *hν* are shown in the inset of Figure 3. The optical energy band gaps *E_g_* determined from the plots are 2.7, 3.0 and 3.2 eV for samples A, B and C, respectively. The main reason for the reduced *E_g_* values in our polycrystalline films as compared with monocrystalline tin dioxide (with *E_g_* ~3.6 eV) is the density of state tails in the forbidden gap due to structural inhomogeneity of the samples.

**Figure 4:**
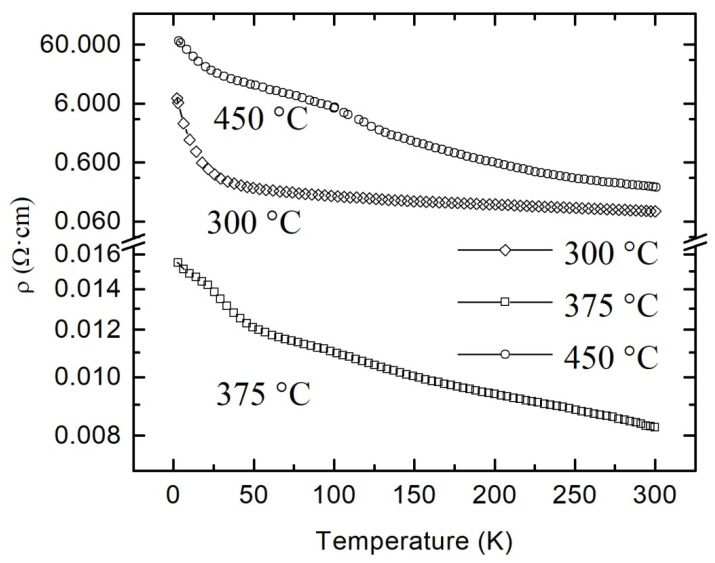
Temperature dependences of the resistivity of polycrystalline SnO_2−δ_ films deposited by reactive magnetron sputtering of tin on glass with the following annealing in air at different temperatures.

The temperature dependences of the resistivity of the films annealed at different temperatures are shown in Figure 4. As one can see, all films exhibit a negative temperature coefficient of the resistance over the whole temperature range of 4–300 K, indicating thus a typical for semiconductors behavior. Samples A and C are characterized by high resistivity at room temperature (9.2 × 10^−2^ and 2.2 × 10^−1^ Ω·cm, respectively) and ratio *ρ*(4 K)/*ρ*(300 K) (~65 and ~295, respectively) in comparison with the film fabricated at 375 °C on the second stage of the annealing procedure. The detailed analysis of the charge transport mechanisms in these more high-ohmic samples, together with their SEM image analysis, will be reported elsewhere.

Taking into account the low value of *ρ*(300 K) and ratio *ρ*(4 K)/*ρ*(300 K) (8.3 × 10^−3^ Ω·cm and 1.9, respectively) for the most conductive sample B, we can conclude that the reactive magnetron sputtering with 1% oxygen content with the further annealing at 375 °C provides close to the optimal conditions for the synthesis of films with the maximum concentration of oxygen vacancies. Estimated from the Hall measurements, the concentration and the mobility of electrons for this film were 2.4 × 10^20^ cm^−3^ and 3.15 cm^2^/V·s, respectively. In tin dioxides films with high charge carriers concentration, the temperature dependences of the resistivity *ρ*(*T*) in the low-temperature range can be interpreted within the framework of quantum corrections to the classical Drude conductivity mechanism [15,26,27,28,29]. It should be noted that usually, WL and EEI effects inherent to the materials characterized by a low value of the temperature resistance coefficient (ratio *ρ*(4 K)/*ρ*(300 K) is usually within the range of about 1–2). Crossover from the typical for the metals positive temperature coefficient of the resistance (*dR*/*dT* > 0) to the negative one (*dR*/*dT* < 0) at the temperature decreasing can also be observed for highly doped semiconductors [36].

*R*(*T*) dependence with a negative temperature coefficient of the resistance (*dR*/*dT* < 0) can also be observed for semiconductors in which the hopping conductivity through the sites in the impurity band (formed due to a high concentration of defects) is responsible for the charge carriers transport. Therefore, we verified this assumption by means of the parameter *W*(*T*) calculation, according to the method proposed by Zabrodskii and Zinovieva [37]:

*W*(*T*) *= d* [ln *σ*(*T*)]*/d* [ln *T*]. (3)

**Figure 5:**
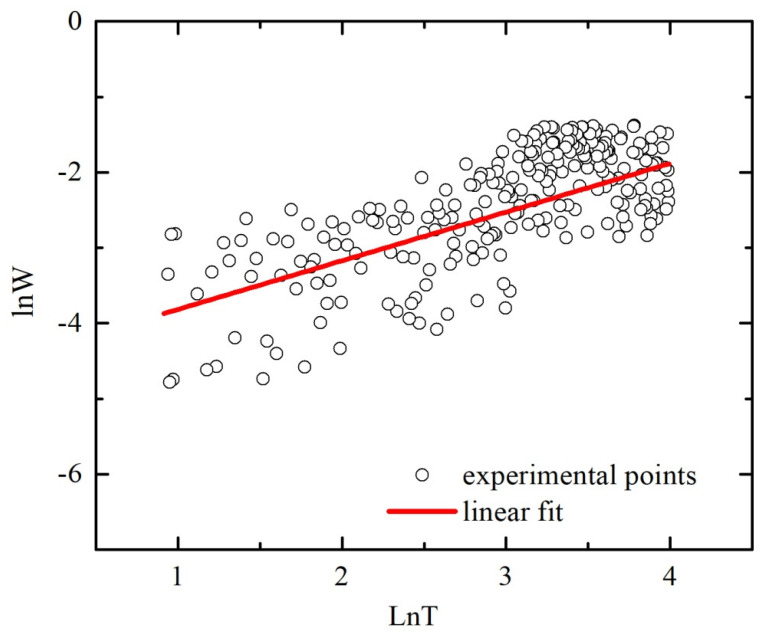
Plot ln*W* versus ln*T* for polycrystalline SnO_2−δ_ film deposited by reactive magnetron sputtering of tin on glass with the following annealing in air at 375 °C (sample B).

A negative slope of ln[*W*(*T*)] vs. ln *T* indicates that the sample is insulating, whereas a positive slope corresponds to the metallic behavior of the sample. Figure 5 shows the plot ln[*W*(*T*)] vs. ln *T* for the most conductive tin dioxide film. The more detailed analysis allows to identify a sample as a real metal (in the case when *W*(*T*) decreases towards zero at the temperature decreasing) or as a weakly insulating (in the case of the temperature-independent behavior *W*(*T*)). In accordance with the criterion [37], the observed positive slope of the ln[*W*(*T*)] vs. ln *T* plot with a value of about 0.65 excludes the possibility to interpret the *ρ*(*T*) dependence as an electron hopping over localized states through the energy levels of the oxygen vacancies.

Thus, we can assume that our sample is a degenerate semiconductor. We used the Mott criterion [38] in order to verify this assumption. According to this criterion, a semiconductor becomes degenerate when the electron concentration *n* exceeds the critical electron concentration *n_c_* that can be determined by the relation:

(4) $$n_{C}^{1/3}a_{B}=0.26,$$

where *a_B_* is the effective Bohr radius: *a_B_* = *4πεε*_0_*ћ*^2^/m*e^2^.

Estimation of the critical electron concentration for similar polycrystalline tin dioxide films gives the value *n_c_* = 2.24 × 10^18^ cm^−3^ in assumption that the static dielectric constant for SnO_2_ *ε =* 11.65 and the electron effective mass m* = m_e_* = 0.31*m*_0_ [26]. This *n_c_* value is much lower than the concentration in our sample B (2.4 × 10^20^ cm^−3^) and confirms that our most conductive sample is a degenerate semiconductor. This fact can explain the weak temperature dependence of the conductivity and the charge carriers concentration in our sample.

Using the free-electron model, the Formula for the Fermi energy *E_F_*:

*E_F_* = *ћ*^2^*k_F_*^2^/(2 m*), (5)

where m* is the electron effective mass, *ћ* is the reduced Planck’s constant, *k_F_* = (3*π*^2^*n*)^1/3^ is the Fermi wave vector, we get *E_F_* = 330 meV. The position of the Fermi level deep in the conduction band means that the grain boundaries can be considered as the effective scattering regions due to the high concentration of defects in them. At the same time, the condition *E_F_* >> *k_B_T* explains the weak temperature dependence of the electron mobility since only carriers near the Fermi level determine the charge transport properties of the sample.

It should be noted that the reliability of the results of Hall measurements for polycrystalline samples with low mobility of charge carriers is still debated [39] with the exception of the boundary cases: (1) at high charge carrier concentration, the role of barriers becomes insignificant, and conductivity is determined by scattering within crystallites; (2) at low charge carrier concentration, when the electrical conductivity is determined by the potential barriers between grains [39].

Taking into account that our sample B is a degenerate disordered semiconductor, we analyzed its electrical properties within the frame of the quantum corrections to conductivity model similar to the tin dioxide films investigated in [15,26,27,28,29].

There is a possibility to determine the dimensionality of disordered systems with respect to the WL and EEI effects by means of their temperature dependences of the conductivity *σ*(*T*) analysis [30,40]. It is known that the induced by WL correction term to the Drude conductivity for 2D disordered systems *Δσ*_2*D-WL*_(*T*)~ln*T*, for 3D disordered systems *Δσ*_3*D-WL*_(*T*)~*T^p/^*^2^, where *p* is defined by the dominant inelastic scattering mechanism (*p* is equal to 3/2, 2, and 3 in the case the of electron–electron scattering in dirty limit, clean limit or electron–phonon scattering, respectively) [30,40,41]. EEI effects have the following type of temperature dependences of the quantum corrections to the conductivity: for 2D disordered systems *Δσ*_2*D-EEI*_(*T*)~ln*T*, for 3D disordered systems *Δσ*_3*D-WL*_(*T*)~*T*^1/2^ [30,40,41].

If the characteristic length parameters, describing WL and EEI (inelastic scattering length *L_φ_* = (*Dτ_φ_*)^1/2^ and thermal length *L_T_* = (*Dћ*/*k_B_T*)^1/2^, respectively, where *D* is the diffusion constant, *τ_φ_* is the inelastic scattering time), exceeds the thickness of the system, the temperature dependence of the resistance follows a behavior inherent for 2D disordered systems [30,40]. Otherwise, the temperature dependence of the conductivity *σ*(*T*) should be described as typical for 3D disordered systems dependences.

The second possibility to determine the dimensionality of WL and EEI effects in a disordered system is the analysis of the magnetoresistance (MR) curves. One of the main features of charge transport in the WL regime is a negative MR. The influence of EEI on the MR of the disordered systems is more complicated. For example, the sign of MR can be changed from negative to positive one at low temperatures and in high magnetic fields when the energy difference between the Zeeman split levels strongly exceeds the thermal energy (*gμ_B_B* >> *k_B_T*) [42].

As far as *σ*(*T*) dependence for the most conductive tin dioxide film (sample B) at low temperatures (*T* < 25 K) can be approximated by logarithmic dependence (*σ*(*T*)~ln*T*), we made an attempt to use valid for 2D disordered systems Hikami formula (neglecting the spin–orbit interaction and the scattering by magnetic impurities) for the approximation of the conductivity on magnetic field dependences *σ*(*B*) [43]:

(6) $$\sigma \left(B\right)=\sigma_{0}+\frac{e^{2}}{2\pi^{2}ћ}\left[\Psi \left(\frac{1}{2}+\frac{B_{2}}{B}\right)-\Psi \left(\frac{1}{2}+\frac{B_{1}}{B}\right)\right]$$

where *Ψ* is the digamma function, *B*_1_ and *B*_2_ are parameters characterizing the processes of elastic and inelastic scattering, respectively defined by the following formula:

*B_x_* = ћ/4*eD*τ_*x*_, (7)

where index *x* = 1 or 2, *D* = *υ_F_*^2^*τ*_1_/2 is the diffusion constant, *τ*_1_ = *l*/*υ_F_* is the elastic scattering time, *l* is the elastic scattering length, *τ*_2_ = *τ_i_* is the inelastic scattering time, *e* is the electron charge, *υ_F_* is the Fermi velocity.

**Figure 6:**
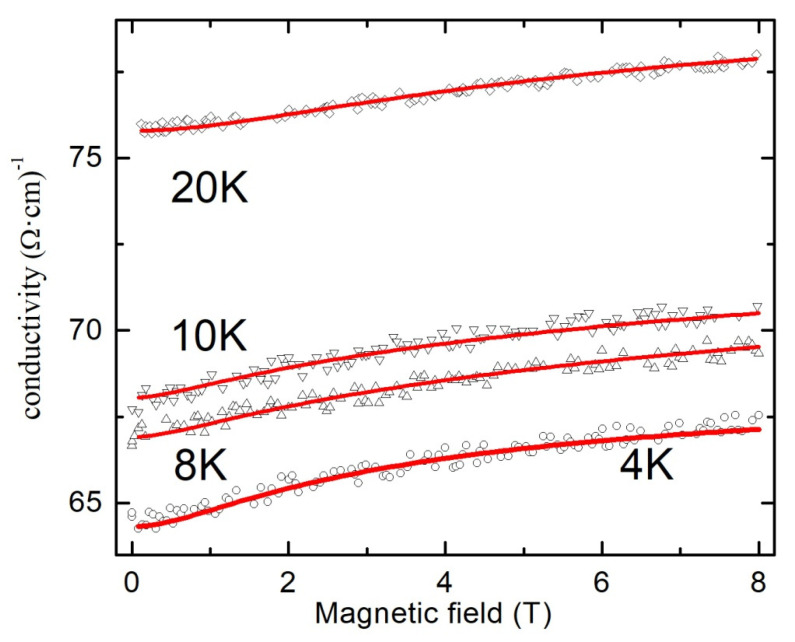
Magnetoconductivity at various temperatures of polycrystalline SnO_2−δ_ film (sample B). Solid lines are fits by Formula (6).

The measured at temperatures 4, 8, 10 and 20 K dependences *σ*(*B*) for the most conductive sample B and their approximation by Formula (6) are shown in Figure 6.

**Figure 7:**
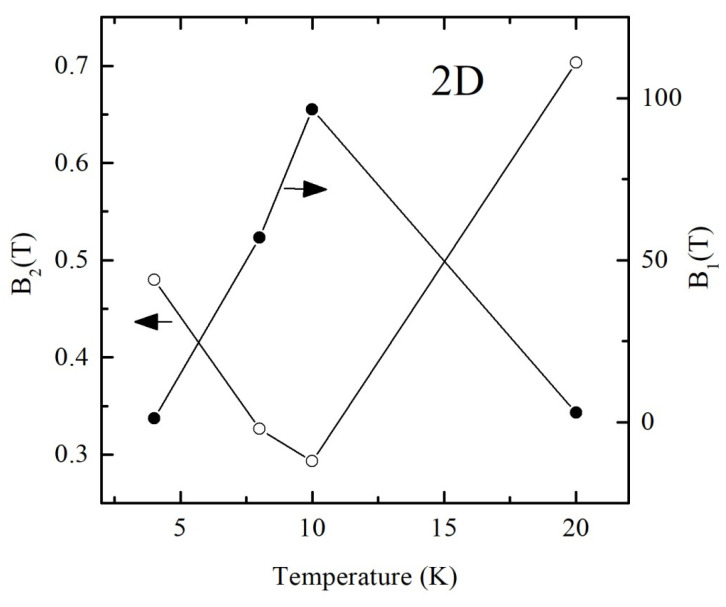
Temperature dependences of the parameters *B*_1_ and *B*_2_ calculated from the fitting curves using Formula (6) for polycrystalline SnO_2−δ_ (sample B).

As one can see, experimental results are in good agreement with the theoretical fits. In order to prove the assumption about the possibility to use valid for 2D disordered system Formula (6) for the approximation of the magnetoresistance data, we calculated the temperature dependences of the parameters *B*_1_ and *B*_2_. These results are shown in Figure 7. The decrease in the value of the parameter *B*_2_ observed in the temperature range 4–10 K with the temperature contradicts the theory since it would mean an increase in the time of inelastic scattering and, as a result, an increase of the negative MR (positive magnetoconductance) with the temperature due to WL effect. A significant increase in the temperature range 4–10 K in the value of the parameter *B*_1_ also does not agree with the theory since it would mean a significant decrease by orders of magnitude in the elastic scattering time and would also enhance the WL effect.

Hence, we can conclude that the approximation of MR results within the frame of the model valid for 2D disordered systems gives the wrong temperature dependences of the parameters *B*_1_ and *B*_2_. Therefore, we use the following formula for 3D disordered systems for analyzing MR data [44]:

(8) $$\frac{\Delta \rho (B)}{\rho_{0}^{2}}=-\frac{e^{2}}{2\pi^{2}ћ}\sqrt{\frac{eB}{ћ}}f_{3}\left(\frac{B}{B_{i}}\right),$$

where *B_i_* = *ћ*/4*eDτ_i_* refers to the inelastic scattering fields, *D* is the diffusion constant, *τ_i_* is the inelastic scattering time. The function *f*_3_(*x*) can be defined as:

(9) $$f_{3}(x)=2\left(\sqrt{2+\frac{1}{x}}-\sqrt{\frac{1}{x}}\right)-\left[{\left(2+\frac{1}{x}\right)}^{-1/2}+{\left(\frac{3}{2}+\frac{1}{x}\right)}^{-1/2}\right]+\frac{1}{48}{\left(2.03+\frac{1}{x}\right)}^{-3/2}$$

**Figure 8:**
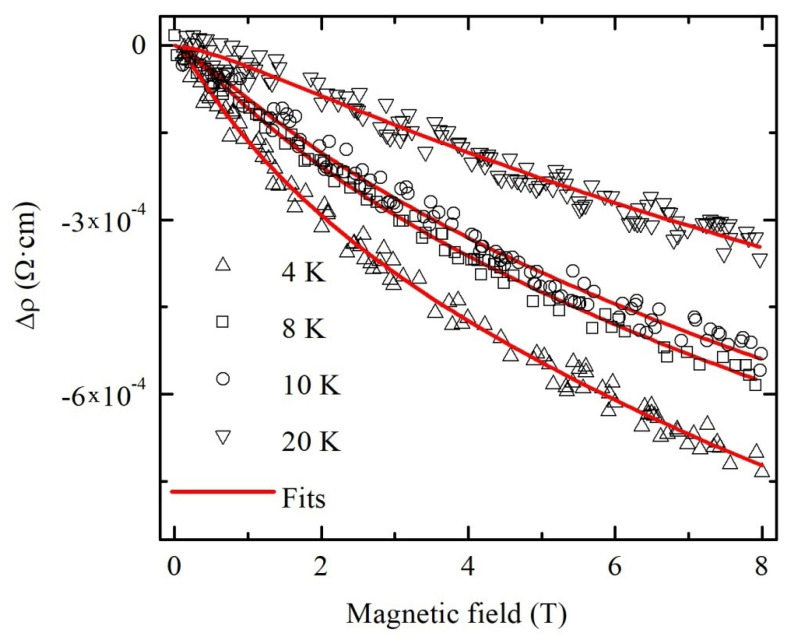
Magnetoresistivity at various temperatures of polycrystalline SnO_2−δ_ (sample B). Solid lines are fits by Formula (8).

The measured at temperatures 4, 8, 10 and 20 K dependences ∆*ρ*(*B*) for the most conductive sample B and their approximations by Formula (8) are shown in Figure 8.

We should emphasize a good correspondence between experimental results and theoretical fits by Formula (8). The values of the parameters *B_i_* calculated from the fitting curves were used for the estimation of electron dephasing length *L_φ_* by means of the following formula:

(10) $$L_{\varphi }\approx \sqrt{D\tau_{i}}=\sqrt{ћ/4eB_{i}}.$$

**Figure 9:**
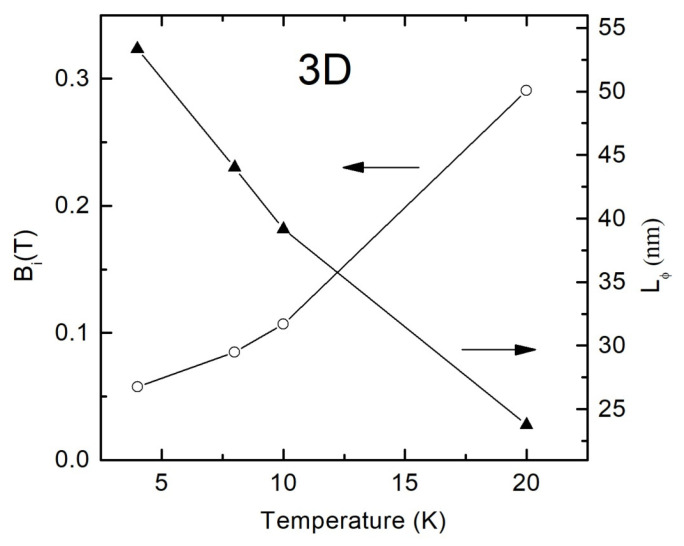
Temperature dependences of the parameters *B_i_* and *L_φ_* calculated from the fitting curves using Formulas (8) and (10) for polycrystalline SnO_2−δ_ film (sample B).

The temperature dependences of the parameters *B_i_* and the electron wave function dephasing length *L_φ_* are shown in Figure 9. As one can see from the figure, even at a minimum temperature of 4 K, *L_φ_* does not exceed 55 nm for the sample under study, which is significantly less than the sample thickness of *d*~100 nm. This fact, together with the theoretical prediction that the phase coherence length decreases with the temperature, allows us to conclude about the 3D dimensionality of our system with respect to the WL effect. The dominant scattering mechanism of charge carriers responsible for the electron phase loss in the WL regime can be determined from the temperature dependence of the phase coherence length *L_φ_*~*T^−p^*^/2^. It is known that parameters *p* = 3 and *p* = 1.5 are attributed to the electron–phonon scattering and to the electron–electron scattering with small energy transfer, respectively [30,40]. However, the small number of experimental points in Figure 9 does not allow to reliably define the value of *p*.

More accurate analysis of the dominant charge carriers scattering mechanism can be done by means of the analysis of the temperature dependence of the conductivity *σ*(*T*) in the low-temperature range in which quantum corrections to the conductivity due to WL and EEI are usually observed.

**Figure 10:**
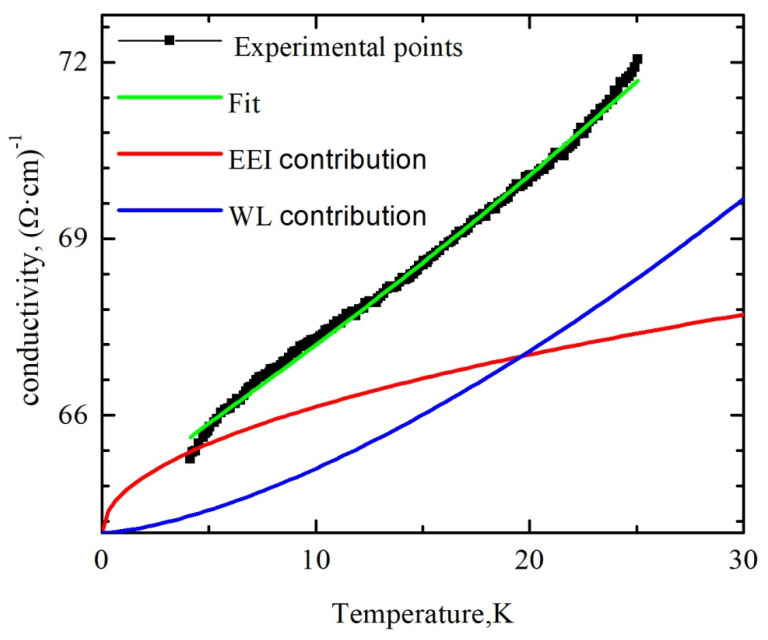
Temperature dependence of the conductivity of polycrystalline SnO_2−δ_ film (sample B). The solid lines are the fits by Formula (11).

As soon as we found, on the basis of the MR data analysis, that our film behaves like a 3D system with respect to the WL effect, we used for the analysis of the *σ*(*T*) formula inherent for the 3D character of quantum corrections to the conductivity. In the general case taking into account corrections both due to WL and EEI effects, the temperature dependence of the conductivity can be written as follows:

*σ*(*T*) = *σ*_0_ + *A·T^p^*^/2^ + *B·T*^1/2^, (11)

where *σ*_0_ is the residual conductivity, the second and the third terms are the corrections from the WL and EEI effects, respectively. As mentioned above, *p* = 3 or *p* = 3/2, in dependence on the dominant scattering mechanism. The better fitting results were obtained for the parameter *p* = 3, indicating that the WL effect due to electron–phonon scattering dominates in our sample. The dependence *σ*(*T*) in the low-temperature range and fitting curve are shown in Figure 10.

Figure 10 also shows the last two terms of the Formula (11) separately shifted for clarity by *σ*_0_. As one can see from Figure 10, the decrease in conductivity of tin dioxide film in the temperature range of 4–25 K occurs mainly due to the contribution of the WL effect, and the contribution from EEI to the quantum corrections to the conductivity is unessential. This fact confirms the eligibility of using for the fitting of the MR data Formula (8), which takes into account 3D WL effects. We assume that EEI effects should be taken into account at lower temperatures and in higher magnetic fields. Investigation of the influence of the tin dioxide film thickness on their temperature dependence of conductivity and magnetoresistance can provide information about peculiarities of the transition from the 3D to 2D nature of the weak localization effect with the decrease in the film thickness.

It should be noted that the electron wave function dephasing length *L_φ_* at low temperatures significantly exceeds the size of the crystallites. Hence, we can assume that the elastic scattering is enhanced at the crystallite boundaries due to nonuniform defect density distribution. Some errors in the determination of the film parameters (dephasing length, mobility) can be induced by nonuniform defect distribution. The decrease in the *L_φ_* value as compared with the data of [26] can be associated with a high density of defects at the grain boundaries and, as a result, with an additional contribution of elastic scattering on the film surface. For example, Hall measurements give a very low mobility value: at *T* = 300 K mobility μ~3.15, ~8–12 and ~4.43–5.89 cm^2^/V·s for our film and for the similar polycrystalline tin dioxide films studied in [26] and in [27], respectively. Therefore, the condition for the observation of WL in disordered systems (*k_F_l* >> 1) is hardly realized.

We can assume that in disordered metal-oxide materials (even under degeneracy conditions), the elastic scattering at the boundaries of crystallites plays a significant role. As a result, the calculated from the Hall measurements mobility value can be much lower than the mobility inside crystallites. This means higher values of the mean free path of electrons and the product *k_F_l*.

## 4. Conclusions

We demonstrated the possibility to synthesize polycrystalline tin dioxide films with the electrical conductivity varied in a wide range of magnitude using the reactive magnetron sputtering with low oxygen content (about 1 vol %) in argon–oxygen plasma with the following treatment at high temperatures in the range of 300–450 °C. The electrical properties and the magnetoresistance of the most conductive sample were studied in detail. It was found that in the low-temperature range (~4–25 K) conductivity of this film is determined by the 3D WL effect with the dominating electron–phonon scattering mechanism.

## References

[1] Batzill M., Diebold U. (2005). The surface and materials science of tin oxide. Prog. Surf. Sci..

[2] Hartnagel H.L., Dawar A.L., Jain A.K., Jagadish C. (1995). Semiconducting Transparent Thin Films.

[3] Barquina P., Martins R., Pereira L., Fortunato E. (2012). Transparent Oxide Electronics: From Materials to Devices.

[4] Pereira M.S., Lima F.A.S., Ribeiro T.S., Da Silva M.R., Almeida R.Q., Barros E.B., Vasconcelos I.F. (2017). Application of Fe-doped SnO2 nanoparticles in organic solar cells with enhanced stability. Opt. Mater..

[5] Leem J.W., Yu J.S. (2011). Physical properties of electrically conductive Sb-doped SnO2 transparent electrodes by thermal annealing dependent structural changes for photovoltaic applications. Mater. Sci. Eng. B.

[6] Kikuchi N., Kusano E., Kishio E., Kinbara A. (2002). Electrical and mechanical properties of SnO2: Nb films for touch screens. Vacuum.

[7] Gordon R. (1997). Chemical vapor deposition of coatings on glass. J. Non-Cryst. Solids.

[8] Shajira P.S., Bushiri M.J., Nair B.B., Prabhu V.G. (2014). Energy band structure investigation of blue and green light emitting Mg doped SnO2 nanostructures synthesized by combustion method. J. Lumin..

[9] Oshima T., Okuno T., Fujita S. (2009). UV-B Sensor Based on a SnO_2_ Thin Film. Jpn. J. Appl. Phys..

[10] Huang H., Tian S., Xu J., Xie Z., Zeng D., Chen D., Shen G. (2012). Needle-like Zn-doped SnO_2_nanorods with enhanced photocatalytic and gas sensing properties. Nanotechnology.

[11] Shankar P. (2015). Gas sensing mechanism of metal oxides: The role of ambient atmosphere, type of semiconductor and gases—A review. Sci. Lett. J..

[12] Das S. (2014). SnO2: A comprehensive review on structures and gas sensors. Prog. Mater. Sci..

[13] Serin T., Yildiz A., Serin N., Yildirim N., Ozyurt F., Kasap M. (2010). Electron-Electron Interactions in Sb-Doped SnO_2_ Thin Films. J. Electron. Mater..

[14] Guillen C., Herrero J. (2019). Intrinsic and extrinsic doping contributions in SnO_2_ and SnO_2_: Sb thin films prepared by reactive sputtering. J. Alloys Compd..

[15] Gao K.H., Lin T., Liu X.D., Zhang X.H., Li X.N., Wu J., Liu Y.F., Wang X.F., Chen Y.W., Ni B. (2013). Low temperature electrical transport properties of F-doped SnO_2_ films. Solid State Commun..

[16] Varley J.B., Janotti A., Van de Walle C.G. (2010). Group-V impurities in SnO_2_ from first-principles calculations. Phys. Rev. B.

[17] Kılıç C., Zunger A. (2002). Origins of Coexistence of Conductivity and Transparency in SnO_2_. Phys. Rev. Lett..

[18] Godinho K.G., Walsh A., Watson G.W. (2009). Energetic and Electronic Structure Analysis of Intrinsic Defects in SnO_2_. J. Phys. Chem. C.

[19] Gürakar S., Serin T. (2019). Comprehensive structural analysis and electrical properties of (Cu, Al and In)-doped SnO2 thin films. Mater. Sci. Eng. B.

[20] Bazargan S., Heinig N.F., Rios J.F., Leung K.T. (2012). Electronic Transport in Tin(IV) Oxide Nanocrystalline Films: Two-Medium Transport with Three-Dimensional Variable-Range Hopping Mechanism for the Ultrasmall Nanocrystallite Size Regime. J. Phys. Chem. C.

[21] Giraldi T.R., Lanfredi A.J.C., Leite E.R., Escote M.T., Longo E., Varela J.A., Ribeiro C., Chiquito A.J. (2007). Electrical characterization of SnO_2_: Sb ultrathin films obtained by controlled thickness deposition. J. Appl. Phys..

[22] Serin N., Yildiz A., Alsaç A.A., Serin T. (2011). Estimation of compensation ratio by identifying the presence of different hopping conduction mechanisms in SnO_2_ thin films. Thin Solid Films.

[23] Muraoka Y., Takubo N., Hiroi Z. (2009). Photoinduced conductivity in tin dioxide thin films. J. Appl. Phys..

[24] Ksenevich V.K., Dovzhenko T.A., Dorosinets V., Melnikov A.A., Wieck A.D. (2008). Electrical Properties and Magnetoresistance of Nanogranular SnO_2_ Films. Acta Phys. Pol. A.

[25] Serin N., Yildiz A., Serin T. (2011). Electrical Properties of Polycrystalline SnO_2_ Thin Films. Appl. Phys. Express.

[26] Li Q.L., Zhang X.H., Lin T., Gao K.H. (2018). Electrical transport properties of polycrystalline SnO_2_ thin films. J. Alloys Compd..

[27] Boyali E., Baran V., Asar T., Ozcelik S., Kasap M. (2017). Temperature dependent electron transport properties of degenerate SnO_2_ thin films. J. Alloys Compd..

[28] Dauzhenka T.A., Ksenevich V.K., Bashmakov I.A., Galibert J. (2011). Origin of negative magnetoresistance in polycrystalline SnO_2_ films. Phys. Rev. B.

[29] Ksenevich V.K., Dauzhenka T.A., Galibert J. (2013). Weak localization and electron-electron interactions in polycrystalline tin dioxide films. J. Phys. Conf. Ser..

[30] Altshuler B.L., Aronov A.G., Efros A.L., Pollak M. (1985). Electron-Electron Interactions in Disordered System.

[31] Adamchuck D.V., Ksenevich V.K. (2019). Control of Electrical and Optical Parameters of Humidity Sensors Active Elements Based on Tin Oxides Films with Variable Composition. Devices Methods Meas..

[32] Boroojerdian P. (2013). Structural and Optical Study of SnO Nanoparticles Synthesized Using Microwave—Assisted Hydrothermal Route. Int. J. Nanosci. Nanotechnol..

[33] Adamchuck D.V., Ksenevich V.K., Poklonski N.A., Navickas M., Banys J. (2019). Nonstoichiometric tin oxide films: Study by X-ray diffraction, Raman scattering and electron paramagnetic resonance. Lith. J. Phys..

[34] Wood D.L., Tauc J. (1972). Weak Absorption Tails in Amorphous Semiconductors. Phys. Rev. B.

[35] Gümü C., Ozkendir O.M., Kavak H., Ufuktepe Y. (2006). Structural and optical properties of zinc oxide thin films prepared by spray pyrolysis method. J. Optoelectron. Adv. Mater..

[36] Liu X.D., Jiang E.Y., Li Z.Q. (2007). Low temperature electrical transport properties of B-doped ZnO films. J. Appl. Phys..

[37] Zabrodskii A.G., Zinoveva K.N. (1984). Low-temperature conductivity and metal-insulator transition in compensate n-Ge. Sov. Phys. JETP.

[38] Mott N. (1982). Review lecture: Metal-insulator transitions. Proc. R. Soc. London. Ser. A Math. Phys. Sci..

[39] Werner F. (2017). Hall measurements on low-mobility thin films. J. Appl. Phys..

[40] Lee P.A., Ramakrishnan T.V. (1985). Disordered electronic systems. Rev. Mod. Phys..

[41] Du G., Prigodin V.N., Burns A., Joo J., Wang C.S., Epstein A.J. (1998). Unusual semimetallic behavior of carbonized ion-implanted polymers. Phys. Rev. B.

[42] Monsterleet J.M., Capoen B., Biskupski G. (1997). The importance of electron interaction to the negative magnetoresistance of metallic n-GaAs close to the metal-insulator transition. J. Phys. Condens. Matter.

[43] Hikami S., Larkin A., Nagaoka Y. (1980). Spin-Orbit Interaction and Magnetoresistance in the Two Dimensional Random System. Prog. Theor. Phys..

[44] Likovich E.M., Russell K.J., Petersen E.W., Narayanamurti V. (2009). Weak localization and mobility in ZnO nanostructures. Phys. Rev. B.

